# Development and evaluation of an online training program based on the O-AMAS teaching model for community pharmacists in the post-COVID-19 era

**DOI:** 10.3389/fpubh.2022.906504

**Published:** 2022-09-23

**Authors:** Bingzheng Shen, Jun Liu, Jiahuan Helen He, Zhanyong Zhu, Benhong Zhou

**Affiliations:** ^1^Department of Pharmacy, Renmin Hospital, Wuhan University, Wuhan, China; ^2^School of Pharmaceutical Science, Wuhan University, Wuhan, China; ^3^Health Service Center, Xianghe Community, Wuhan, China; ^4^Department of Physiology, McGill University, Montreal, QC, Canada; ^5^Department of Plastic Surgery, Renmin Hospital, Wuhan University, Wuhan, China

**Keywords:** community pharmacists, continuing education, O-AMAS teaching model, flipped classroom, post-COVID-19 era

## Abstract

**Background:**

Formerly, the community pharmacists' work was mainly focused on drug supply. However, during the COVID-19 epidemic outbreak, community pharmacists in Wuhan played an important role in control and prevention of SARS-CoV-2 and in providing pharmaceutical care. Due to a lack of adequate knowledge and skills, many community pharmacists were not able to cope with healthcare work timely and efficiently. To improve community pharmacists' specialized knowledge and enhance their professional competence through systemic training in the post-COVID-19 era.

**Methods:**

Based on the O-AMAS (Objective, Activation, Multi-learning, Assessment and Summary) teaching model and flipped classroom, an online continuing training program containing four sections was developed. It was a semi-experimental study with no control group. Quantitative tests before and after training as well as questionnaire were used to evaluate the outcome of this training program for community pharmacists.

**Results:**

A total of twenty-six community pharmacists were invited to participate in continuing education, and twenty-five trainees finished this training program with a completion rate of 96.2 %. Quantitative tests before and after training and anonymous questionnaires were carried out to comprehensively evaluate the outcomes of this training program. Compared with the test scores before training (61.6 ± 6.6), the score after training was statistically higher, reaching 80.9 ± 7.5 (*P* < 0.001). Twenty-three questionnaires were received (returns ratio, 92.0%). Notably, most of the pharmacists were satisfied with the training program. The percentage of positive responses for each item in this anonymous questionnaire was more than 85 %.

**Conclusion:**

It was suggested that the O-AMAS model and the flipped classroom-based continuing educational program achieved the expected training effects. It is a promising on-the-job training approach for pharmacy continuing education. Moreover, our study also demonstrated that online learning had advantages of no geographic constraints, flexible learning beyond time and easy interaction, over traditional face-to-face training style, especially in the post-pandemic era.

## Introduction

The pandemic caused by Corona Virus Disease 2019 (COVID-19) was first reported at the end of 2019 in Wuhan City, China (1). On March 11^th^ 2020, after careful evaluation of clinical symptoms and transmission kinetics, the World Health Organization (WHO) characterized it as a global pandemic, and it is the first global pandemic caused by coronavirus termed as severe acute respiratory syndrome coronavirus 2 (SARS-CoV-2) (2). Till now, the variants of SARS-CoV-2, such as Delta and Omicron, are still spreading around the world and affecting our daily life (3, 4).

Since the COVID-19 pandemic outbreak, community pharmacists in Wuhan together with other health professionals have been working on the frontline to fight against the disease. Besides the medication supply, prescription-checking and drug consultation, community pharmacists have been providing reliable information, timely updating patient records, and educating the public on disease preventive methods (5). Furthermore, they have been actively involved in early virus tests, free home delivery services, disease management and carrying out government epidemic prevention strategies, for the sake of slowing down the spread of virus transmission (6–10).

Because of the limited knowledge of the SARS-CoV-2 virus, as well as the lack of adequate clinical pharmacotherapy skills, many community pharmacists often were faced challenges in carrying out anti-epidemic work smoothly. The best way to solve this problem is to develop continuing education and on-the-job training for them. The O-AMAS teaching model, consisting of five key parts (Objective, Activation, Multi-learning, Assessment and Summary), is an effective teaching model presented by Effective Teaching Group, Nankai University, China in 2020 (11, 12), which draw on the strategy of outcomes-based education (OBE) (13–15). We designed a continuing training program based on the O-AMAS model combined with flipped classroom and remote online teaching platform.

This training program included both theoretical knowledge and clinical practice skills, mainly involving drug treatment of infection disease, management and drug therapy of chronic disease, rational use of drugs in special populations and pharmacy practice skills. Compared to the traditional in-person training, the advantage is that the online learning offers more flexibility in time and space for community pharmacist. Based on the case and video, trainers explain every key point in detail and trainees were encouraged to ask any questions at any time in and out of on-line class. Participants can get answers in and after class through online teaching platform. The overall aim of this study was to describe the design, development, implication and outcome evaluation of this online O-AMAS teaching model and flipped classroom-based continuing training program. We hope to establish a new continuing education model for community pharmacists and provide a reference for the design and implementation of on-the-job training in the post-epidemic era.

## Methods

### Participants

This continuing training program was a semi-experimental study with no control group. The training program information was released through “WeChat App,” a social media platform. All community pharmacists working in Xianghe community were encouraged to take part in this training program voluntarily. The exclusion criteria were: 1. non-community pharmacist, 2. currently not employed, 3. <1 year of community pharmacy service experience, 4. inability to use an internet-connected computer or smartphone.

### Training program design

**Figure 1:**
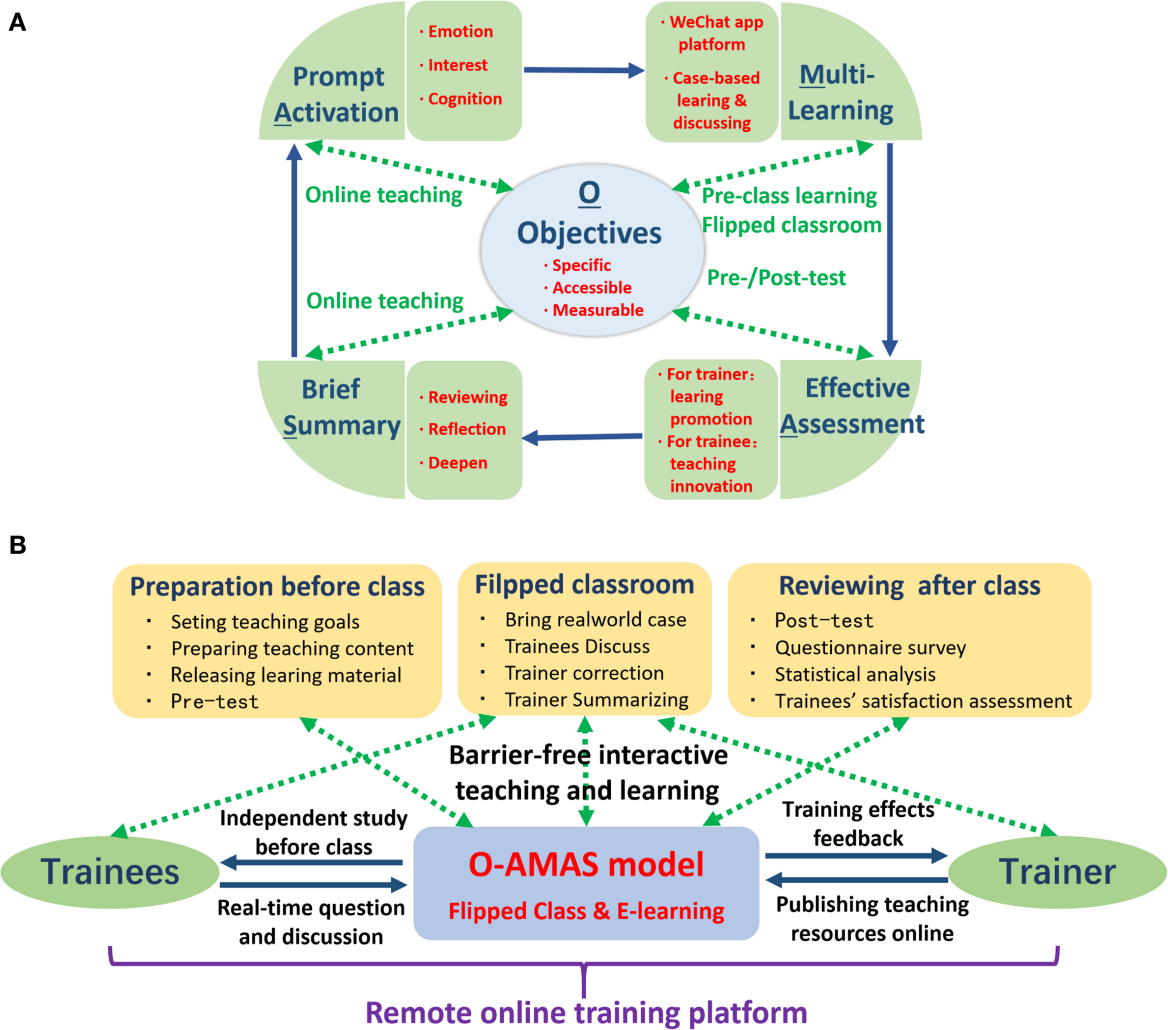
The design and development of the continuing training program for community pharmacists. **(A)** Overall design based on the O-AMAS model and flipped classroom. **(B)** Flow diagram for the organization of the training program.

The O-AMAS effective teaching model was used for the design and development of this training program (11, 12), which was constituted of five modules (Figure 1A). The training effectiveness was the key to this online training for community pharmacists. The trainee's enthusiasm can be activated through emotion, interests and cognition arousal (16). Multiple forms of learning, such as pre-class study, case analysis and online discussion, were applied. Appropriate survey and quantitative test can facilitate teaching and learning. The summary of contents in class can help trainees deepen their understanding, and the case-based teaching and discussion would further improve their abilities to analyze and solve practical problems. The ultimate objective was to realize methodical training and effective learning.

In traditional continuing training for pharmaceutical education, the activities always employed a teacher-centered, lecture-based approach that focused on knowledge impartment rather than application (17). To obtain enough credits, trainees had to passively receive knowledge. The curriculum content they learned can't be integrated into their own knowledge system and flexibly applied to the community health service. To overcome these disadvantages, we adopted a novel pedagogical approach that combined the O-AMAS model and the remote flipped classroom *via* an online teaching platform (18–20). The flow diagram for the training organization was displayed in Figure 1B.

The training contents were arranged according to the service characteristics of community pharmacists and the need for the prevention and control against COVID-19 in the post-epidemic era. The specific teaching contents were listed in Table 1. The pre-class learning materials, including a 5- to 15-page PDF document and videos, would be distributed to the trainees through the WeChat app at least 1 week in advance. The Tencent meeting platform was used for this remote online training. The trainer firstly outlined the theoretical knowledge and explained the difficult points to trainees, and then case-based discussion was carried out to strengthen their ability to cope with practical problems. Since the program was an on-the-Job training, the schedules were arranged in the evening or on weekends. Moreover, all the training videos were recorded so that the training contents can be played back and freely reviewed at any moment.

**Table 1:** The training contents for community pharmacists.

**Sections**	**Training contents**	**Self-learning materials before training**		**On-line training**			**Video recording** ** and playback**
		**PDF/** **page**	**Video/** **min**	**Theoretical** ** training/** **min**	**Number** ** of training** ** case**	**Case** ** discussing/** **min**	
Section 1 Pharmacotherapy in Infectious Diseases	Rational use of anti-bacterial drugs	10	—	30	3	45	Yes
	Rational use of anti-fungal drugs	6	—	30	2	40	Yes
	Rational use of anti-viral drugs	8	—	20	2	40	Yes
Section 2 Management and pharmacotherapy of chronic diseases	Appropriate blood pressure control and rational use of antihypertensive drugs	12	—	30	2	60	Yes
	Rational use of drugs for regulating blood sugar	11	—	30	3	60	Yes
	Rational use of lipid-regulating drugs	10	—	30	2	45	Yes
	Rational use of anticoagulants	9	—	30	2	40	Yes
**Mid-term Q&A time**	Answer trainees' questions related to Chapters 1 and 2 (30~60 min)	—	—	—	—	—	No
Section 3 Pharmacotherapy for special population	Rational drug use for pregnant and lactating women	15	—	40	3	60	Yes
	Rational drug use for the elderly	13	—	20	3	40	Yes
	Rational drug use for people with liver or kidney insufficiency	10	—	30	4	60	Yes
Section 4 Practical skills	Cardio-pulmonary resuscitation	6	15	10	1	15	Yes
	Heimlich maneuver	5	10	10	1	15	Yes
	Wound treatment and dressing	6	12	15	2	20	Yes
	Emotion and stress regulation	6	8	15	2	20	Yes
Final Q&A time	Answer trainees' questions related to the whole training program (60~90 min)	—	—	—	—	—	No

### Evaluation of training effects

All trainees were asked to provide basic personal information *via* WeChat (21). The pre- and post- tests were conducted in order to quantitatively measure the training effects, and these two tests adopted the same type of exam question possessing the same difficulty. The test consisted of three parts: single-answer question, multiple-answers question and case-analysis question (Supplementary Table 1). To better assess the trainees' problem-solving competence in their practical work, the test focused on case analysis, which the score accounted for 70 %.

Trainees would receive an electronic questionnaire form at the end of this training program. To get their real feelings about this program and encourage truthfulness, their personal identifiable information was not collected within the questionnaire. Each of the survey items in the questionnaire was a single best answer question, which can avoid trainees giving it up due to cumbersome way as much as possible. The contents of the questionnaire were displayed in Table 2.

**Table 2:** Questionnaire of continuing training program for community pharmacist.

**Survey item**	**Agree**	**Neutral**	**Disagree**
1. Compared with the traditional teaching method, I prefer this training strategy.	**□**	**□**	**□**
2. I have better learning experience and classroom participation.	**□**	**□**	**□**
3. It is easier to understand the knowledge points.	**□**	**□**	**□**
4. This training program improved my vocational capability to solve practical problems.	**□**	**□**	**□**
5. Discussion on real-world cases helps me to think as actively as the health worker.	**□**	**□**	**□**
6. Flipped classroom allows me to clarify learning contents before class.	**□**	**□**	**□**
7. Remote online teaching can save me a lot of time on traffic.	**□**	**□**	**□**
8. The function of video playback in the platform allow me to review the course or re-arrange my training time if there is a time conflict.	**□**	**□**	**□**
9. The training platform based on internet, WeChat app and Tencent meeting platform facilitate the interaction between trainees and trainers.	**□**	**□**	**□**
10. I hope that other training programs can also adopt this teaching strategy in the future.	**□**	**□**	**□**

### Statistical analysis and data management

All measured variables were displayed as means ± SD for continuous variable. For comparisons of test scores before and after training, a two-tailed Student *t*-test for paired test was used to assess the differences of the data by GraphPad Prism version 9.0 software. A *P*-value < 0.05 would be considered statistically significant.

After the trainees successfully registered for our training project, an independent and unique ID number was automatically assigned to every trainee *via* online teaching platform. All trainers and researchers identify different trainees only by their ID numbers. The trainees' personal information and data were managed and protect through two people two passwords strategy. Moreover, in order to achieve anonymous test and survey, the ID number was set to be invisible by the administrator.

## Results

### Characteristics of trainees

A total of twenty-six community pharmacists were recruited and received this continuing training program willingly *via* an on-line training platform. Only one of them didn't finish all the required training courses. Therefore, the results were calculated and analyzed based on the remaining twenty-five trainees. The overall completion rate for our continuing educational program was 96.2 %. The basic socio-demographic features for the trainees were shown in Table 3. The ratio of female trainees was in line with the percentage in the global healthcare workforce (22). On account of the continuous investment in community medical hardware and huge improvements in their incomes, most pharmacists had bachelor degrees or above and possessed the primary and middle professional titles. More than half of the trainees had <5 years of community pharmacy work experience.

**Table 3:** Social-demographic profile of all twenty-five trainees.

**Characteristic**	**Category**	**Frequency,** ***n***	**Percentage,** ** %**
Gender	Male	7	28.0
	Female	18	72.0
Age	21~30	13	52.0
	31~40	9	36.0
	41~50	2	8.0
	51~60	1	4.0
Educational level	Junior college	3	12.0
	Undergraduate	19	76.0
	Master	3	12.0
	Doctor	0	0.0
Professional title	Primary	8	32.0
	Middle	14	56.0
	Vice-senior	2	8.0
	Senior	1	4.0
Years of working in pharmacy service	0~5	14	56.0
	6~10	8	32.0
	11~20	2	8.0
	>20	1	4.0

### The analysis of pre- and post-test scores

**Figure 2:**
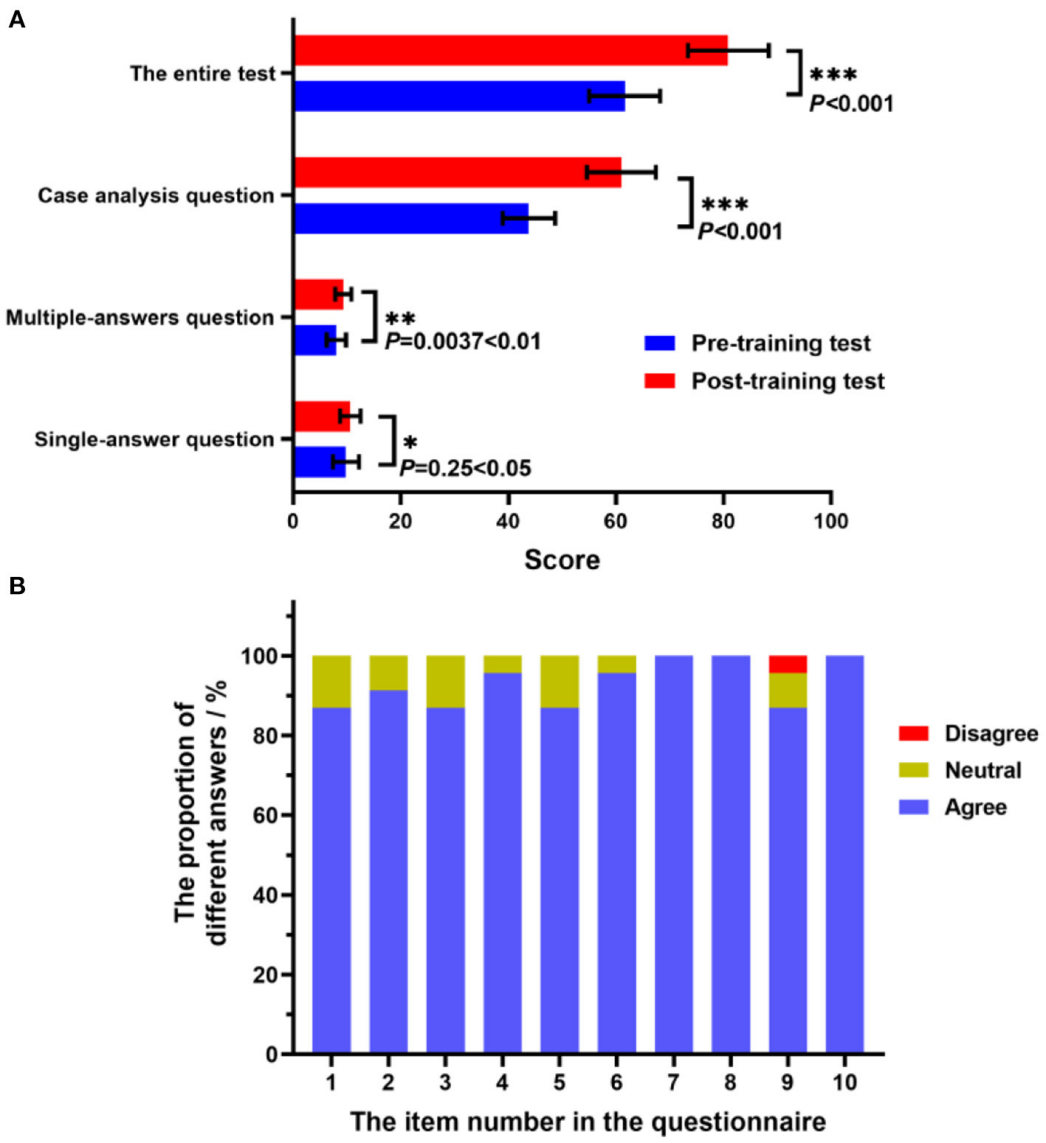
Analysis of pre- and post-training test score and questionnaires. **(A)** The total score and the scores of each question type before and after training. ^*^P < 0.05, ^**^P < 0.01, ^**^P < 0.001 vs Pre-training test. **(B)** The percentage of each option for every item in questionnaire.

Quantitative scores of pre- and post-training tests were collected to analyze trainees' learning outcomes. Totally, twenty-five community pharmacists who finished all required items were involved and assessed. The results were displayed in Figure 2A. The comparison of the total test scores before and after training (61.6 ± 6.6 and 80.9 ± 7.5, respectively) showed a significant improvement with *p* < 0.001. The scores of each question type, involving single-answer question, multiple-answers question and case-analysis question, were also statistically analyzed. After receiving this training program, the scores of every question type were had been increased, and the differences were statistically significant (*p* < 0.05). Especially, for the case-analysis question, compared to the pre-test score of 43.8 ± 4.9, the trainees obtained a much higher score of 61.0 ± 6.4 after receiving this on-the-job training (*p* < 0.001). It was suggested this program could help trainees enhance their ability to deal with complex pharmacy service.

### Questionnaire

The online questionnaire survey was carried out to assess trainees' attitudes on their learning experiences and satisfaction degree *via* WeChat. The questionnaire contents focused on the training methods for community pharmacists, mainly including the flexibility, convenience, accessibility, efficiency and practicality of the training program. The survey was not mandatory. In all the twenty-five learners who had completed this training, twenty-three valid questionnaires were received. The effective rate of returned questionnaires was 92%. The statistical results of all survey items in e-questionnaire described previously were revealed in Figure 2B. More than 85 % of trainees gave positive comments on each question in the questionnaire. And the ratio of neutral responses ranged from 4 to 14 %. It was indicated that most of them could accept the training approach and contents. For questions 7, 8 and 10, all trainees chose the option “agree.”

## Discussion

Community pharmacists played an irreplaceable role and made substantial contributions to the fight against COVID-19 (23). During the pandemic outbreak period (January to April, 2020), the community pharmacists working in Wuhan city felt that their theoretical knowledge and practical skills were insufficient. The aim of developing this training program was to enhance their professional competence. An effective teaching model named O-AMAS was adopted to design the entire framework and organize the contents of the training program. The two key points of this model are realization of teaching objectives and good learning experiences. The O-AMAS model emphasizes that the training should focus on the outcome after receiving the program. Objective-oriented lecture was always used in continuing education to pursue excellent and effective training. If trainees felt welcomed, included and valued, they would put their heart and soul into learning (24). Therefore, trainers should maintain positive interaction with trainees during the whole process of their training.

The flipped classroom, one of the most popular teaching methods, has been widely used in higher education courses (25), but it is rarely used in the continuing training for pharmacists. Compared to traditional learning, the trainees were required to learn the basics themselves before attending training program. The trainer was seen as a helper and a facilitator rather than the installer of knowledge. The transformation of trainer's role, case-based training and trainees' acceptance were the three key elements to successfully leverage the benefits of flipped classroom. Owe to the on-the-job training, all adult trainees had limited study time. As shown in Table 1, the pre-class study material was <15 pages. After refinement and modification, the cases from the real-world practice of community pharmacy were used for this training, which can cover the learning contents and improve trainees' ability to solve practical problems. The results of the questionnaire reflected their acceptance of this training strategy. Although the flipped classroom pedagogy can bring many advantages to improve learning performance, some challenges, such as inadequate self-learning before training, increasing study load and more time consuming, need to improve.

The emergence of SARS-CoV-2 and its variants challenged the traditional continuing training system. The closure of classrooms and unsafe traveling could lead to a lot of troubles and accelerate the spread of COVID-19. Thanks to the progress in information technology and the rise of mobile internet, the on-line training can be carried out smoothly. The remote online training can fully meet the requirements of epidemic prevention and control policy. It was trainee-centered and provided more flexibility in terms of location and time. For the program developers, the online training also enabled them to utilize a great number of online tools, involving text, motion graph, audio and video, to create an efficient teaching and an intelligible learning environment. Without any barrier, the online training platform combined with flipped classroom methods can establish an interactive and collaborative training atmosphere where all trainees are happy to ask questions freely, provide immediate feedback. Although online continuing education showed many advantages in the post-pandemic era, there were still some problems to be solved (26). It was worth noting that one trainee chose the “disagree” option for question 9 in questionnaire “The training platform based on internet and Wechat app facilitate the interaction between trainees and trainers?” (Figure 2B). The most intractable issue might be that the older trainees can't expertly use the internet and online training platform. For this, necessary technical assistance and support should be provided to a few trainees. In consequence, outcome-based quality management and continuous improvement are the two critical points for the success of online continuing training.

Through receiving this continuing training, trainees were expected to achieve these four-level goals: remembering, understanding, applying and creating. These four-level goals were drawn according to Blooms' Taxonomy (27, 28), a classic approach of training goals classification, multidimensional cognition, affection and psychomotor abilities. This training program, combining the advantages of the O-ASAM teaching model, flipped classroom and online education, improved community pharmacists' occupational competency.

Compared to traditional small-scale offline training, it is a time- and place-free program without limitation of maximum number of trainees. In addition, previously, most programs for community pharmacists were related to specific vocational skills, such as mental health care (29), chronic disease management (30, 31) and pharmacy administration (32). However, there was few on-the-job trainings focused on the improvements of their professional abilities to deal with public health events and outbreaks of virulent infectious diseases. This online program provides relatively systematic training contents, including theoretical knowledge and practical skills.

Despite this online training program presented many advantages, there are still some limitations and shortcomings need to refine. Trainees in traditional physical classrooms can always discuss puzzling questions about learning with others. Furthermore, body languages including facial and hand gestures are absent in on-line training. The lack of physical interactions would have a negative impact on the effectiveness of the learning and hinder the training process (33). For trainers, the omission of trainees' emotions may prevent them from responding to community pharmacist's needs.

## Conclusion

An online continuing training program, combined O-AMAS effective teaching model and flipped classroom strategy, for community pharmacists was designed and developed. The results of quantitative testing and questionnaire survey suggested that the program described in this study displayed a successful way to update professional theory and enhance pharmaceutical care skills. In addition, the innovative way of thinking in pharmacy service they learned from this program may be very useful for their future study and career development.

From this study, we found that the professional competency of competencies of community pharmacy staff is insufficient. It is urgent to strengthen the basic knowledge of clinical diseases and improve their first-aid operations. The number of community pharmacists recruited in this study was limited. We will expand the sample size to further evaluate this continuing training program.

## Data availability statement

The original contributions presented in the study are included in the article/Supplementary material, further inquiries can be directed to the corresponding authors.

## Ethics statement

The studies involving human participants were reviewed and approved by Ethics Committee, Health Service Center, Xianghe Community. The patients/participants provided their written informed consent to participate in this study.

## Author contributions

BS, JL, and BZ conceived the study and designed the method. BS, JL, and ZZ performed the applied teaching model. BS, JL, JH, and BZ contributed to the analysis and interpretation of the data and provided important scientific input. BS, JH, and BZ wrote and revised the manuscript. All authors discussed the results, contributed to the final manuscript, contributed to the development, and approved the final manuscript.

## Funding

This work was supported by the Research Grants for Teaching Reform of Wuhan University School of Medicine (2021084 and 2019026 to BS), the Research Funding of China Association of Higher Education (21JXYB06 to ZZ). The funders had no role in study design, data collection and analysis, preparation of the manuscript or decision to publish.

## Conflict of interest

The authors declare that the research was conducted in the absence of any commercial or financial relationships that could be construed as a potential conflict of interest.

## Publisher's note

All claims expressed in this article are solely those of the authors and do not necessarily represent those of their affiliated organizations, or those of the publisher, the editors and the reviewers. Any product that may be evaluated in this article, or claim that may be made by its manufacturer, is not guaranteed or endorsed by the publisher.
