# Supplementary material for: Development and evaluation of an online training program based on the O-AMAS teaching model for community pharmacists in the post-COVID-19 era
Source: Front Public Health. 2022 Sep 23;10:906504. doi: 10.3389/fpubh.2022.906504 (PMC9538181; doi:10.3389/fpubh.2022.906504)
Supplement: Supplementary file 1 [file Table_1.DOC]

**Supplementary Table 1**

The type, quantity and score of each question in pre- and post-training tests

| Training contents | Single-answer question | | Multiple-answers question | | Case analysis  question | |
| --- | --- | --- | --- | --- | --- | --- |
|  | Quantity | Score | Quantity | Score | Quantity | Score |
| Chapter 1 | 4 | 4 | 2 | 4 | 2 | 20 |
| Chapter 2 | 6 | 6 | 2 | 4 | 2 | 20 |
| Chapter 3 | 4 | 4 | 2 | 4 | 2 | 20 |
| Chapter 4 | 2 | 2 | 1 | 2 | 1 | 10 |
